# Umbilical cord management in newborn resuscitation

**DOI:** 10.1038/s41390-024-03711-5

**Published:** 2024-11-11

**Authors:** J. S. Dorling, C. C. Roehr, A. C. Katheria, E. J. Mitchell

**Affiliations:** 1 Neonatal Medicine, University Hospital Southampton NHS Foundation Trust, Southampton, UK; 2 NIHR Southampton Biomedical Research Centre, Faculty of Medicine, University of Southampton, Southampton, UK; 3 National Perinatal Epidemiology Unit Clinical Trials Unit, University of Oxford, Oxfordshire, UK; 4 Newborn Care, Southmead Hospital, Bristol, UK; 5 Faculty of Health Sciences, University of Bristol, Bristol, UK; 6 Neonatal Research Institute, Sharp Mary Birch Hospital for Women and Newborns, San Diego, CA USA; 7 Neonatal Research Institute, San Diego, CA USA; 8 Nottingham Clinical Trials Unit, Faculty of Medicine and Health Sciences, University of Nottingham, Nottingham, UK

At birth, blood continues to flow from the mother to baby through the intact umbilical cord. This continuum of fetal-placental circulation, now taking place between the placenta and the newborn infant has been termed ‘placental transfusion’, and it enables redistribution of blood between the placenta and the baby.^1^ There is usually net flow into the baby, which appears to play a role in expanding pulmonary blood.^2,3^ Ample clinical evidence has shown that uninterrupted placental transfusion, as supported by the practice of deferred umbilical cord clamping (DCC) reduces death, especially in preterm babies who do not require resuscitation at birth, i.e., those born in “good condition”. In an individual patient data meta-analysis (48 trials, 6367 babies <37 weeks’ GA), DCC compared to immediate clamping reduced death before discharge by almost a third (odds ratio [OR] 0.68 95% confidence interval [CI] 0.51–0.91).^4^ Accepted clinical practice is, therefore, to defer cord clamping in stable term and preterm infants whenever possible. Current international newborn resuscitation guidelines recommend at least 60 seconds of DCC.^5^ Moreover, in a sub-set of the recent individual patient data meta-analysis (47 trials, 6094 babies), deferring cord clamping beyond 120 seconds further reduced the odds of death (OR 0.31 Cl 0.11–0.80).^6^ We speculate these recent new insights will be considered in the next edition of resuscitation guidelines.

Aside from the considerable body of evidence in stable term and preterm infants, very few trials have investigated the groups of babies who needed resuscitation at birth. It is crucial, therefore, that the above results are not extrapolated to the population of compromised babies without adequate evidence. In addition, as most studies were unable to accurately record who needed resuscitation, it is challenging, retrospectively, to reliably extract data on intervention and control groups.

In this edition of Pediatric Research, Major and co-authors present a meta-analysis (6 trials, 539 babies) of trials that explicitly reported on all, or nearly all babies who were resuscitated with an intact cord (“intact cord resuscitation”).^7^ Sample sizes ranged from 37 to 162 babies per trial and most trials were conducted in high-income countries. Four trials included babies below 32 weeks’ gestational age, one included babies over 32 weeks and one babies of greater than 33 weeks’ gestational age. In their analyses, Major et al. highlight the lack of evidence for important outcomes: there were no statistically significant differences for in-hospital mortality, oxygen saturation level, Apgar scores, temperature at NICU admission, or early complications of prematurity. The small sample size of the studies included, and inadequate power for most of the outcomes, is likely to explain these findings. There was a suggestion that intact cord resuscitation may improve oxygenation at 5 minutes of age, as this parameter nearly reached statistical significance, when data from the three trials reporting it were combined. The mean difference in saturation of oxygen at 5 minutes was 6.67% (95% Cl –1.16–14.5).^7^

Recognizing the lack of evidence for intact cord resuscitation at the time of writing (in 2020/2021), current international resuscitation guidelines^8,9^ highlighted the need for more evidence of the benefit of DCC for babies who need resuscitation. Consequently, there appears to be a recent, increasing enthusiasm for practicing DCC in babies who need resuscitation. Various approaches and equipment have been developed to provide resuscitation with the cord intact.^10–13^ Blood transfused from the placenta may provide oxygen, blood cells and fluid volume, all of which may be beneficial during and after immediate resuscitation.^9,12,14^ Resuscitating with the umbilical cord intact could therefore potentially save lives and prevent brain and other vital organ injury, and, later, disability. However, intact cord resuscitation is without doubt technically more challenging than DCC alone as it requires additional skilled staff and new or adapted equipment. Furthermore, consideration for the parents needs to be made, as all resuscitative actions are seen/witnessed by the mother and birthing partner, who then can have immediate interaction with the baby and neonatal team.^10,13,15^ This may enhance communication and parental understanding of the actions taken during resuscitation, which could improve parental psychological outcomes, although close involvement might also be traumatic, requiring careful debriefing and follow-up.^15–17^

To conclude, and as highlighted by the systematic review by Major et al.^7^ the safety and efficacy of intact cord resuscitation remains an important, unanswered research question. Preventing just a few cases of disability per thousand liveborn could substantially improve parental quality of life and save considerable resources for healthcare and society.^18,19^ Due to the lack of evidence on when to clamp the cord in the very vulnerable population of compromised babies at birth, the question of whether to resuscitate with the umbilical cord intact, or not, was voted the second most important question in a recent UK neonatal research priority setting exercise.^18^

An adequately powered, large randomized controlled trial is needed to guide clinical practice for these newborn babies.
